# Psychoactive drug advertising: a comparison of technical information from three countries: Brazil, United States and United Kingdom

**DOI:** 10.1590/S1516-31802005000500002

**Published:** 2005-09-01

**Authors:** Patricia de Carvalho Mastroianni, José Carlos Fernandes Galduróz, Elisaldo Araujo Carlini

**Keywords:** Pharmaceutical preparations, Propaganda, Advertising, Psychotropic drugs, Legislation, Preparações farmacêuticas, Propaganda, Publicidade, Psicotrópicos, Legislação

## Abstract

**CONTEXT AND OBJECTIVE:**

Studies carried out in the 1970s and 1980s showed that there were country-dependent disparities in the information given for the same drug in medical advertisements. National and international regulations have been published to do away with such disparities and to foster the rational use of drugs. The purpose of this study was to compare the information contained in psychoactive drug advertisements published in psychiatric journals in Brazil, the United States and the United Kingdom, before and subsequent to the publication of the United States Export Act, in 1986, the WHO criteria, in 1988, and the Brazilian Sanitary Surveillance Agency Resolution no. 102, in 2000.

**TYPE OF STUDY AND SETTING:**

Content analysis, at Centro Brasileiro de Informações sobre Drogas Psicotrópicas (Cebrid).

**METHODS:**

We gathered advertisements from Brazilian, American and British psychiatry periodicals published before and after each ruling. We analyzed a total of twenty-four Brazilian advertisements that were for the same psychoactive drugs as advertised in American and/or British publications from the same period.

**RESULTS:**

We observed that Brazilian advertisements omitted information on usage restrictions, such as contraindications, adverse reactions, interactions, warnings and precautions, and that such information was present in American and British advertisements.

**CONCLUSIONS:**

The data suggest that disparities in the information given for the same drug still persist. The information depends on the country in which each drug is marketed. The legislation is insufficient for eradicating such disparities.

## RESUMO

**CONTEXTO E OBJETIVO:**

Trabalhos realizados nos anos 70 e 80 demonstraram que havia diferenças de informações para um mesmo medicamento dependendo do país em que era comercializado. Regulamentos nacionais e internacionais foram publicados com o objetivo de extinguir tais diferenças e fomentar o uso racional de medicamentos. O objetivo deste trabalho é comparar as informações contidas nos anúncios de medicamentos psicoativos publicados em periódicos de psiquiatria brasileiros, norte-americano e ingleses, antes e após a publicação do “Export Act”, publicado em 1986 nos Estados Unidos; dos “Critérios da WHO”, em 1988, e a Resolução da Diretoria Colegiada n^o^ 102, de 2000, da Agência Nacional Vigilância Sanitária do Brasil.

**TIPO DE ESTUDO E LOCAL:**

Análise de conteúdo realizado no Centro Brasileiro de Informação sobre Drogas Psicotrópicas (Cebrid).

**MÉTODOS:**

O conteúdo dos anúncios foi analisado de acordo com técnica de análise de conteúdo, segundo as exigências dos regulamentos. Ao todo, foram analisados 24 anúncios brasileiros de medicamentos psicoativos em comum com os anúncios americanos e/ou ingleses publicados no mesmo período.

**RESULTADOS:**

Observou-se que os anúncios brasileiros omitem informações que restringem o uso dos remédios como contra-indicações, reações adversas. interações, advertências e precauções sobre os medicamentos, e estas mesmas informações estão presentes em anúncios norte-americanos e ingleses.

**CONCLUSÕES:**

Os dados deste trabalho sugerem que persistem até hoje as diferenças de informações de um mesmo medicamento em anúncios, dependendo do país de comercialização e que as legislações não são suficientes para extingui-las.

## INTRODUCTION

Non-ethical drug advertising is a severe problem in most of the world, but mainly in developing countries, and can result in irrational use of medication, over-prescribing, self-medication and abuse.^1,2^

The main purpose of drug advertising regulations is to improve healthcare through the rational use of medications,^3^ so as to ensure that, in using the information contained in advertisements, doctors will not produce a negative outcome for their patients.

With this in mind, in 1986, the United States Congress enacted the Export Act,^4^ through which United States multinationals were ordered to include abroad the same information as enforced domestically through approval from the Food and Drug Administration (FDA).

The Act stipulated that the labeling for drugs sold in countries such as Brazil and other developing countries should be the same as approved for such drugs under the Public Health Service Act and the Federal Food, Drug and Cosmetic Act. Furthermore, it determined that any labeling differences should be based on valid scientific evidence, including clinical investigations.

Up to that time, drugs that were not approved in the United States by the FDA could be exported and traded in other countries such as Brazil.^4^

With the same objective, the World Health Organization (WHO) published its “Ethical and scientific criteria for drug advertising” in 1988. This document was intended to regulate drug advertising aimed at professional doctors and the public, as well as the distribution of free samples, pharmaco-vigilance, the disclosure of information, the content of directions/prescriptions and labels, and the conduct of advertisers.^3–5^

Among the recommendations in the document, it was stated that advertisements for prescribed drugs should at least include the following information: generic name, trade name, indications, dose and presentation, name of excipients that could trigger known problems, adverse reactions, precautions, contraindications, warnings, principal interactions, name and address of the manufacturer or distributor, and officially acknowledged references.^3^ These requirements from WHO are recommendations to member countries and do not have the force of law over the State.

In Brazil, there was already a regulation for drug advertising through Law 6,360,^6^ of 1976, stipulating that “text, figures, images or projections shall not insinuate false interpretations, error or confusion regarding the composition of the product, its purpose, mode of use or origin, or advertise therapeutic properties not proven at the time of registration”, and that “contraindications, indications, precaution, and warnings regarding the use of the product must be declared”.

Law 9,294,^7^ of 1996, supported the same ethical and scientific criteria as put forward by WHO in 1988 but was only officially implemented four years later through ANVISA (*Agência Nacional de Vigilância Sanitária*, the Brazilian sanitary surveillance agency), through the publication of Resolution no. 102,^8^ on November 30, 2000, thereby approving the drug advertising regulations.

Drug advertising plays an important role in choosing the drug to be prescribed. Advertising is the main source of fast information for doctors, who take an average of just five minutes to obtain information on the drugs they prescribe, because of their work overload, through working for 50 to 60 hours per week.^9^

Advertisements in specialized medical journals play a significant role in publicizing psychoactive drugs, in that the advertising of prescription drugs is only for the benefit of the professionals who prescribe and dispense them.^3,8^

Assessment of the advertising for psycho-active medication is relevant, since this drug class is the third largest in Brazil in terms of numbers of prescriptions.^10^ Furthermore, studies carried out in the 1970s and 1980s showed that the advertising for psychoactive drugs tended to be less informative than the advertising for other therapeutic classes.^11,12^

Studies analyzing and comparing the directions and compendia for medications in developed and developing countries have identified differing patterns of information for the same drug from the same multinational.^4,13,14^ Although such differences have been observed with regard to directions and compendia, little has been said about the differences in information regarding drug advertising, especially in Brazil.

## OBJECTIVE

The purpose of this study was therefore to compare the information contained in the advertising for psychoactive drugs published in Brazilian psychiatric journals with the advertising for the same medications published in American and British journals, before and after the Export Act of 1986,^4^ the WHO criteria of 1988^3^ and ANVISA Resolution no. 102^8^ of 2000 were published.

## METHODS

### Sample

We gathered advertisements for psychoactive drugs from psychiatric journals containing drug advertising that were indexed within the National Library of Medicine system (List of Journals Indexed in Index Medicus, 1999),^15^ and within the Latin American and Caribbean health sciences literature system (*Literatura Latino-Americana e do Caribe de Ciências da Saúde*, LILACS)^16^ (Pan-American Health Organization, PAHO/WHO, 1997).^17^ Indexed journals offer the advantage of easy access to database research and are therefore the most widely used and most accessible in several university libraries. Furthermore, they ensure periodicity of publications for fact finding. The Brazilian journals that fulfilled the criteria established were: Arquivos de Neuropsiquiatria, Revista de Psiquiatria Clínica, Jornal Brasileiro de Psiquiatria and Revista Brasileira de Psiquiatria.

The selection criterion adopted for the Brazilian advertisements was that they should relate to the same drugs as advertised in publications from the United States and United Kingdom. We chose to analyze American periodicals because the Export Act^4^ was enacted by the United States Congress. The periodicals analyzed were the American Journal of Psychiatry and Archives of General Psychiatry. British periodicals were selected because the advertisements were in the same language as the American ones, and because they represented a developed country from Europe. The British periodicals analyzed were the British Journal of Psychiatry and Journal of Neurology Neurosurgery & Psychiatry.

We selected advertisements for psychoactive drugs published during two distinct periods:

- **December 1985 to May 1989** - a period going from one year before to one year after both the American Export Act^4^ and the WHO ethical and scientific criteria, taken to be a single period of study because the advertisements were the same;
- **December 1999 to December 2001** - a period going from one year before to one year after ANVISA Resolution no. 102,^8^ of 2000, which also presented the same advertisements before and after the ruling.

### Gathering, organizing and identifying advertisements

We started by collecting advertisements from Brazilian publications. We then collected advertisements published in American and British periodicals relating to drugs containing the same active ingredients and from the same laboratory, regardless of whether the brand name was the same as in the Brazilian advertisement.

We analyzed 118 issues of Brazilian journals and 266 issues of American and British journals. We thus obtained advertisements for 24 drugs that were common to the Brazilian and English-language publications.

After organizing the advertisements for the psychoactive drugs, they were then analyzed in detail for their content. Content analysis is defined as a technique for the treatment of research data that aims to objectively, systematically and quantitatively describe the “communication” content. This technique is used to analyze texts and interviews, among other source materials. Thus, although originating from quantitative research, content analysis requires interpretation of materials of a qualitative nature.^18^

The analysis began by scanning through the advertisements. Scanning is the analysts’ first contact with the study material, and it has the aim of establishing an impression and the guidelines for the material.^19^ It will indicate the direction to be taken in preparing a plan for the content analysis.

### Script for advertisement content analysis

We drew up a script for the advertisement content analysis based on the regulatory requirements,^4,8^ in order to individually analyze the advertisements. The script took the following into account:

- Part I - **Identification:** to identify what the drug was (trade name, generic name, laboratory and therapeutic class) and where it was advertised (Brazilian, American or British periodicals).
- Part II Part II - **Technical content**: to identify and quantify the information on indications, dosage, posology, adverse reactions to medications, interactions, precautions, and warnings, with regard to: a) whether present; b) number of items of information per technical item; and c) emphasis on each technical item.
- Part III Part III - **General characteristics**: with regard to figures, catch-phrases and possible irregularities present in the advertisement. Information was regarded as noncompliant when prohibited under the regulations, as were any and all statements that were subjective, or that did not present officially acknowledged references.

### Analysis

The Brazilian, American and British advertisements were compared for the presence of, average amount of information on, and emphasis placed on technical items in each study period.

## RESULTS

We analyzed advertisements for 24 psychoactive drugs marketed in Brazil that were common to Brazilian, American and/or British psychiatry publications. For the period from 1985 to 1989, we found 9 drugs advertised both in Brazil and in the United States or United Kingdom. We gathered advertisements for 15 drugs advertised from 1999 to 2001 (Table 1).

**Table 1.** Psychotropic medication advertised in psychiatric journals* from Brazil, the United States and/or the United Kingdom, analyzed according to therapeutic class

Therapeutic class	Period I (December 1985 to May 1989)	Period II (December 1999 to December 2001)
**Antidepressives**	Ludiomil® (maprotiline) Tofranil® (imipramine)	Aropax^®^/Seroxat^®^ (paroxetine) Cipramil^®^ (citalopram) Effexor XR^®^ (venlafaxine) Luvox® (fluvoxamine) Prolift^®^ (reboxetine) Remeron^®^ (mirtazapine) Zoloft^®^ (sertraline)
**Neuroleptics**	None	Geodon^®^ (ziprasidone) Leponex^®^ (clozapine) Risperidal^®^ (risperidone) Seroquel^®^ (quetiapine) Solian^®^ (amisulpride) Zyprexa^®^ (olanzapine)
**Anxiolytics**	Buspar® (buspirone) Frontal®/Xanax® (alprazolam) Lorax^®^ (lorazepam)	None
**Hypnotics**	Tranxene^®^ (clorazepate) Halcion^®^ (triazolam)	Sonata^®^ (zaleplon)
**Anticonvulsants**	Tegretol^®^ (carbamazepine)	Topamax^®^ (topiramate)
**Antiparkinsonians**	Prolopa^®^/Madopar^®^) (levodopa plus benserazide)	None

* Revista Brasileira de Psiquiafria; Jornal Brasileiro de Psiquiafria; Revista de Psiquiafria Clinica; Arquivos de Neuropsiquiafria; American Journal of Psychiatry; Archives of General Psychiatry; British Journal of Psychiatry; Journal of Neurology Neurosurgery & Psychiatry.

During the analysis, we observed the ratio between the number of pages of advertising and the number of pages of text. This ratio was found to be around 1:5 for the Brazilian publications, 1:4.5 for American publications and 1:20 for British publications.

There were no significant differences between the periods analyzed. All of the advertisements for the drugs analyzed, irrespective of nationality of the periodical, featured the name of the active ingredient and the name of the laboratory. The Brazilian advertisements presented the least number of technical items, especially in relation to information on usage restrictions such as contraindications, adverse reactions, interactions and precautions (Table 2). They were also less informative than the American or British advertisements, particularly with regard to adverse reactions and precautions (Table 3).

**Table 2.** Percentage of technical information included in the advertisements gathered in the years 1985 through 1989 and 1999 through 2001 in psychiatric journals* from Brazil (BR), United Kingdom (UK) and United States of America (USA)

Technical information	Period I (December 1985 to May, 1989)			Period II (December 1999 to December 2001		
	BR n (%)	USA n (%)	UK n (%)	BR n (%)	USA n (%)	UK n (%)
**Generic name**	**9** (100.0)	**7** (100.0)	**3** (100.0)	**15** (100.0)	**10** (100.0)	**12** (100.0)
**Indication**	**8** (88.8)	**7** (100.0)	**3** (100.0)	**13** (86.6)	**10** (100.0)	**12** (100.0)
**Posology**	**7** (77.7)	**4** (57.1)	**3** (100.0)	**10** (66.6)	**4** (40.0)	**12** (100.0)
**Presentation**	**9** (100.0)	**6** (85.7)	**3** (100.0)	**12** (80.0)	**9** (90.0)	**12** (100.0)
**Laboratory**	**9** (100.0)	**7** (100.0)	**3** (100.0)	**15** (100.0)	**10** (100.0)	**12** (100.0)
**Precautions**	**6** (66.6)	**7** (100.0)	**3** (100.0)	**10** (66.6)	**10** (100.0)	**12** (100.0)
**Contraindication**	**6** (66.6)	**7** (100.0)	**2** (66.6)	**10** (66.6)	**10** (100.0)	**12** (100.0)
**Adverse reaction**	**6** (66.6)	**7** (100.0)	**3** (100.0)	**10** (66.6)	**10** (100.0)	**12** (100.0)
**Interaction**	**5** (55.5)	**7** (100.0)	**3** (100.0)	**9** (60.0)	**10** (100.0)	**12** (100.0)
**Reference**	**2** (22.2)	**2** (28.5)	**2** (66.6)	**9** (60.0)	**7** (70.0)	**5** (41.6)
**Noncompliance**	**2** (22.2)	**0** (0.0)	**0** (0.0)	**5** (33.3)	**3** (30.0)	**0** (0.0)
**Total number of advertisements analyzed**	**9** **(100.0)**	**7** **(100.0)**	**3** **(100.0)**	**15** **(100.0)**	**10** **(100.0)**	**12** **(100.0)**

* Revista Brasileira de Psiquiatria; Jornal Brasileiro de Psiquiatria; Revista de Psiquiatria Clinica; Arquivos de Neuropsiquiatria; American Journal of Psychiatry; Archives of General Psychiatry; British Journal of Psychiatry; Journal of Neurology Neurosurgery & Psychiatry.

**Table 3.** Mean information on each technical item included in the advertisements gathered from psychiatric journals*, from the years 1985 to 1989 and 1999 to 2001, from Brazil (BR), United Kingdom (UK) and United States of America (USA)

Technical information	Period I (December 1985 to May 1989)			Period II (December 1999 to December 2001)		
	BR Mean	USA Mean	UK Mean	BR Mean	USA Mean	UK Mean
**Indication**	4	2	2	2	2	2
**Precautions and warnings**	7	14	6	8	15	10
**Contraindication**	4	4	3	2	2	4
**Adverse reaction**	13	44	13	12	67	28
**Interaction**	6	5	3	5	12	5
**Reference**	2	4	4	6	2	5
**Technical words**	370	550	200	376	2,035	363
**Total number of advertisements analyzed**	**9**	**7**	**3**	**15**	**10**	**12**

* Revista Brasileira de Psiquiafria; Jornal Brasileiro de Psiquiafria; Revista de Psiquiafria Clinica; Arquivos de Neuropsiquiafria; American Journal of Psychiatry; Archives of General Psychiatry; British Journal of Psychiatry; Journal of Neurology Neurosurgery & Psychiatry.

The American advertisements for the same psychoactive drugs featured greater numbers of technical items: they were more informative and consisted of a greater number of words. For instance, they presented five times as many examples of adverse reactions as did the Brazilian advertisements (Table 3).

When these drugs were advertised in British psychiatric journals, the advertisements were more complete and presented all of the technical items required within the WHO criteria (Table 2).

Bibliographic references were the least common item in the advertisements, but were found more often in the second study period (Table 2).

One-third of the references in Brazilian advertisements were from sources not acknowledged officially, or from magazines such as materials from the laboratory itself, articles presented in congresses, oral presentations in seminars or other meetings directed towards specialty areas. Half of the references in the American advertisements were from materials from the laboratory itself, while all of the references in the British advertisements were from indexed periodicals.

The statements that were regarded as not complying with regulatory criteria were: “does not interact with any drug”; “caters to the requirements of all the patients”; “8,000,000 patients treated worldwide”; “The greatest success in the U.S. and a leader in terms of prescription in 13 countries”; and the claim that the drug was indicated for when “peace of mind and simplicity” were required. In addition, one drug omitted information on drug interaction, while vouching for “less risk of drug interaction”.

Another difference found in the advertising in the different countries was the way in which they presented the summarized directions. The British advertisements always presented the directions as footnotes; the American advertisements had them on the next page, as an attachment; and the Brazilian advertisements generally had them on a page attached to the ad or at the end of that issue of the journal.

Brazilian advertisements emphasized information pertaining to clinical indications, presentation, posology and the laboratory's name, with the text in larger print than for the information on contraindications, adverse reactions, drug interactions, precautions and warnings. American advertisements emphasized information on indications, with some information on contraindications, adverse reactions and warnings. In the British advertisements, all of the information was in the same size print, with the exception of the indications, which were given the same emphasis as the brand name of the product.

## DISCUSSION

Studies carried out in the 1970s and 1980s showed that publicity material in developing countries featured large numbers of nonspecific, irrational indications, for which the list of minimum risks and adverse reactions was not even included.^20–23^

The results from the present study have shown that information on usage restrictions, adverse reactions, drug interactions, contra-indications, warnings and precautions was less frequent in Brazilian advertisements than in advertisements in the United States and United Kingdom. When such information was included at all, it was incomplete and in a print size that was smaller than for the items favoring the use of the drug, such as clinical indications, presentation and posology. All of these points make the advertisements less scientific and informative in nature. Drug publicity often omits information on posology, warnings and precautions for the senior citizen age bracket, whereas these are the patients for whom psychotropic drugs are most often prescribed. In the United States, senior citizens are wrongly assigned one third of all the anxiolytics prescribed and half of all the drugs for inducing sleep.^24,25^

The least frequent item for both periods studied proved to be the bibliographic references. This shows that advertisements for psychoactive drugs are more prone to contain marketing and less scientific language, using messages regarding autonomy, satisfaction and fulfillment.^26^

In the United States, only half of the drug advertisements sent to the FDA included references and less than half of these references (43%) were available.^27^ Carandag and Moulds^28^ analyzed the flaws in the information contained in drug advertisements in four Australian medical journals and observed that 15% of the references were cited incorrectly.

One of the most common problems found by the French agency that controls advertising (*Agence Française de Sécurité Sanitaire des Produits de Santé*) is the inclusion of non-indexed references.^29^

A similar study by an international group examined advertisements in higher-circulation medical journals in ten European countries and eight developing countries. This study analyzed 5,711 advertisements in fifteen different medical journals in European countries and 1,199 advertisements in eight medical journals in developing countries. Notable disparities were found in relation to the items of contraindications, warnings and collateral effects, with this information included in over 50% of the European advertisements and in less than 30% in advertisements in developing countries.^5^

Although the present study did not analyze the information content in each reference included in the advertisements, other studies have shown that such irregularities are the ones most often encountered by the agencies responsible for assessing drug advertising before it is published. Studies that evaluated published advertising material have identified scientific articles with no objective; references not published; difficult access to the scientific articles cited; incorrect statistical data sets; incorrect methodologies; bias in the results; unproven experimental data; omission of unfavorable reports; omission of failure rates; faulty information on the outcomes from use; selection of some precautions and warnings, and omission of some data from the original directions; inclusion of unnecessary information such as approval by the FDA; suggested doses that are incoherent with the clinical studies cited; dangerous conclusions, extrapolations and percentage forecasts from small samples; definitions of safety and effectiveness, and suggestions of superiority described in a vague form; and directions on pages differing from the site of the advertisement.^29–38^

Finally in our analysis, we also verified that the ratio between the number of pages of advertisements and number of pages of text was 1:5 for the Brazilian publications; 1:4.5 for American publications, and 1:20 for British publications. Madridejos et al.^39^ assessed the advertising content of Spanish medical periodicals and found that approximately 30% (1:3.3) of the pages contained advertising. This was deemed excessive and suggestions were made for adjustments to international publishing criteria.

## CONCLUSION

The data suggest that disparities in the information given for the same drug in medical advertisement still persist, and that the information given depends on the country in which this drug is marketed. Furthermore, the legislation is insufficient for eradicating such disparities.
